# COVID-19 disaster response: South African disaster managers’ faith in mandating legislation tested?

**DOI:** 10.4102/jamba.v13i1.1099

**Published:** 2021-09-06

**Authors:** Olivia Kunguma, Alice Ncube, Mosekama O. Mokhele

**Affiliations:** 1 Disaster Management Training and Education Centre for Africa, Faculty of Natural & Agricultural Sciences, University of the Free State, Bloemfontein, South Africa

**Keywords:** COVID-19, disaster management, disaster management legislation, disaster managers

## Abstract

For the first time in the history of the Disaster Management Act, 57 of 2002, South Africa declared COVID-19 an epidemiological disaster. Section 3 and 27(1) of this Act activated the responsible Minister in consultation with other Ministers to issue regulations in response to the disaster. The declaration exposed the already criticised Act to scrutiny by the public. Therefore, this study investigated the Metropolitan Disaster Management Centres that coordinate local events and support the provincial and national disaster management centres, their perceptions concerning the disaster management legislation that mandates them. The study recognised a gap in this regard and saw it imperative to give the disaster managers a voice and a platform to express their opinion concerning the heavily criticised legislation. A model of the policy implementation process guided the study investigation. This model argues that implementation of policies tends to generate tensions, which result in a disruption of the policy formulators’ expectations. The research uses some of the model’s variables to measure the perceptions of disaster managers. Using an interview guide, the researchers conducted virtual interviews with the disaster managers. Scholarly and media articles review concerning the Act formed part of the data collection. The study finds that the disaster managers perceive the disaster management legislation as a very useful guide, an excellent piece of legislation and trust it regardless of the criticism it received. The gaps the critics identified in the legislation became evident and had negative effects on the COVID-19 disaster response.

## Introduction

There has been a rise in disasters globally and locally. Worthy of note is the increase in the global and local declaration of epidemiological health-related disasters. Some of the recent disasters identified by the World Health Organisation (WHO) are the Middle East respiratory syndrome coronavirus in 2017, Southern Africa Cholera outbreak in 2018, Middle East respiratory syndrome coronavirus in 2019 and currently the 2020 China respiratory syndrome coronavirus, COVID-19, that was declared a global emergency (WHO 2020a).

As WHO declared COVID-19 a global pandemic, governments started preparing and responding to the pandemic by declaring disasters or emergencies (WHO 2020b). The response to the pandemic became a litmus test for nations and their country’s readiness to deal with the alarming levels of its spread and severity. Following the global declaration, the President of South Africa was one of the first in Africa to declare COVID-19 a national disaster on 15 March 2020 (The Presidency 2020).

For the first time in the history of the South African Disaster Management Act 57 of 2002 (As amended 16 of 2015) (DMA), the South African government activated Section 27 to declare the epidemiological disaster (CoGTA 2020). The President and his Cabinet members imposed a significant number of regulations and restrictions guided by the DMA to curb the spread of the virus. The mandating legislation and the supporting Minister of Cooperative Governance and Traditional Affairs (CoGTA) came under scrutiny by the public. Because of its adoption, the DMA was criticised by various scholars for its failures to curb the intended and untended consequences of disasters, which COVID-19 is not an exceptional case. This concurs with Van Niekerk and Du Plessis (2020) view, ‘COVID-19 shows that old sins have long shadows’. Given the existing challenges facing the DMA, COVID-19 contributed more to these inadequacies of the DMA, which the public raised their views on its implementation towards curbing the spread of the virus (Rabkin 2020).

Nevertheless, a search for publications concerning the disaster managers’ perceptions about the legislation post-COVID-19 revealed limited information. Accordingly, this research saw it imperative to explore the disaster managers’ perceptions regarding the legislation that mandates their operations amidst the public critique. According to Smith (1973), in his Model of the Policy Implementation Process (MPIP), institutions are usually established when a policy is formulated (Wang 2020). Therefore, in the case of the promulgated DMA, the disaster managers are expected to manage the disaster management centres and implement the legislation. Because the disaster managers’ role is to coordinate a disaster as guided by the legislation, the study investigated the disaster managers’ perception of the legislation. This article aimed to contribute to the future review of the disaster management legislation and improve the disaster management operations in an epidemiological disaster and others.

## Before COVID-19: Scholars analysis of the legislation

Because of the promulgation of the DMA and the NDMF, South Africa anticipated that they would holistically guide the prevention and mitigation of social, environmental, political and economic losses during disasters and to provide for effective response and rehabilitation (Sithole 2014). Years later after their promulgation, several scholars analysed and critiqued the legislation revealing deficient implementation processes (Brazer 2018; Harrison 2009; Humby 2012; Jordaan 2018; Pelling & Holloway 2006; Van Niekerk 2014).

Van Niekerk (2014), Humby (2012) and Harrison (2009) mentioned that the DMA is silent on the application of disaster management at the local municipalities. Harrison (2009) also revealed that the DMA’s allocation of powers and duties within the local sphere is limiting. Van Niekerk (2014) further stated that the lack of focus at the local level leads to the avoidance of responsibilities. Brazer (2018) argued that because of a high prevalence of incidents at the local level, the government should shift disaster management focus to the local level. Harrison (2009) further argued that the DMA’s funding focus is only on recovery and rehabilitation contradicting the anticipated proactive nature of the legislation. Pelling and Holloway (2006) observed that the DMA, Section 56(4) states that the organs of state are expected to contribute to disaster management but Section 56(2) conversely states that organs of state ‘may’ financially contribute to response efforts and post-disaster recovery and rehabilitation. Harrison (2009) further indicated that Section 56(2) contravenes with Section 10(a) of the Municipal Systems Act 32 of 2000 that states that an organ of state ‘must’ take appropriate steps to ensure sufficient funding and capacity building initiatives as may be needed for the performance of the assigned function or power by the municipality. Consequently, the use of the terminology ‘may’ and ‘must’ in some sections of the DMA impacts accountability.

Van Niekerk (2014) found that the problematic placement of the disaster management function creates tension and affects the authority to implement the DMA. Concerning the classification and declaration of disasters, the DMA encourages decentralisation, but it is the National Disaster Management Centre’s (NDMC) role to first undertake classifications before the lower-level government can declare a disaster. For classifications to be effortless, the DMA prescribes the development of uniform standards for the assessment of a potential disaster. From the years the DMA and the NDMF were promulgated, the uniform standards were not developed, eventually, the standards were developed in 2016 (Jordaan 2018). Within that context, Van Niekerk (2014) was of the view that the lack of these guidelines caused ambiguity at the various levels of government. In Humby’s (2012) findings, the DMA fails to provide capacity assessment directives on disaster classification and disaster declaration.

Candidly, Humby (2012) divulges how the DMAs legal obligations are counter-productive in the fact that it fails to show phased approaches to the development of disaster management plans. This is also concerning the disaster management plans integration between sectoral strategic plans. Humby (2012) also realised the DMAs failure to specify the allocation of roles between state and non-state disaster management role-players in detail.

Jordaan (2018) also uncovered that the DMA provision to decentralise institutional structures poses challenges for the NDMC by taking away their authority over the other spheres. Van Niekerk (2015) revealed that other stakeholders the disaster managers work with do not understand the DMA and the function. This deficiency was brought to light during the COVID-19 pandemic. Similarly, the DMA is also explicit on its call for the establishment of disaster risk reduction (DRR) focal points in all ministries (Van Niekerk 2015). Because of the promulgation of the DMA, focal points are lacking and the impact of this deficiency in the ministries was observed during the COVID-19 pandemic.

Pelling and Holloway (2006) supported the location of the Disaster Management Function at the highest level of executive authority (Section 1.2.1 of the NDMF) (Republic of South Africa 2005), but the DMA lacks the power and authority to implement the mandate. Moreover, the DMA prescribes that the disaster management function should be established as a department of state under some Minister (Section 8(2)), at the provincial level, under a Member of the Executive Council (MEC) (Section 31(1)). These scholars argue that the current location of the function as a line function limits its authority and has a constrain on cross-sectoral integration. Whilst the DMA strongly requires the transversal mainstreaming of disaster management in organs of state at all spheres of government, it further lacks the powers to implement the mainstreaming (Pelling & Holloway 2006).

Brazer (2018) revealed that the DMA and its redress, the Disaster Management Amendment Act, 16 of 2015 do not provide for Incident Command Systems and multi-agency response to incidents or disasters. Brazer (2018) postulated that a multi-agency response approach is important because incidents or disaster management requires an inter-governmental, inter-organisational and multi-disciplinary stakeholder approach. The COVID-19 pandemic might benefit from such a system. Jordaan (2018) criticised the DMA on the exclusion of the South African National Defence Force (SANDF) as a key stakeholder. He also argues that the omission could be attributed to the prominent role it played under the Civil Defence Act. The fact that the promulgation of a disaster management act was a paradigm shift from civil protection, which was under the SANDF, could have been the reason why the institution was emitted in the DMA.

That said, in 2015, the DMA Amendment Act included the SANDF. This stakeholder (SANDF) played a critical role during the COVID-19 disaster, providing safety and security support during the COVID-19 disaster declaration (Jordaan 2018; Parliamentary Monitoring Group 2020).

## Public critique of the legislation after the COVID-19 disaster declaration

During the disaster declaration, the South African President mentioned that a national state of disaster was declared in terms of the DMA. His mention and acknowledgment of the DMA, as an instrument used to declare the global pandemic COVID-19 a disaster, show that the legislation is powerful (The Presidency 2020). However, the 21-day country lockdown that was imposed as of 26 March 2020 and later lapsed beyond the prescribed days gave room for the public to scrutinise the legislation. One of the strategies criticised was the National Coronavirus Command Council (NCCC) established by the President to coordinate the response to the COVID-19 pandemic (De Vos 2020). Consequently, the powers that the members of this NCCC exercised under the DMA were dubbed sweeping and tested the strength and resilience of South Africa’s constitutional democracy violating human rights (Padayachee et al. 2020; Singh 2020). The President confirming that there was no legal framework used to establish the council revealed its illegitimacy (Head 2020). Interesting to know is the President’s disregard of the activation of the Intergovernmental Committee on Disaster Management (ICDM) as prescribed in the DMA, Section 4(1) (Republic of South Africa 2002; Van Niekerk & Du Plessis 2020), which is constitutional, unlike the NCCC. Alternatively, the South Africans National Health Act, 61 of 2003 also empowers the country’s health Minister to appoint an advisory and technical committee (Singh 2020). Therefore, the Department of Health could have also established such a constitutional body in partnership with CoGTA. However, the Health Act was considered inadequate to deal with COVID-19 (Karrim 2020).

Besides the council, the infamous DMA, and the Minister in charge, Dr Nkosazana Zuma- Dlamini was also publicly and legally scrutinised. The DMA’s deficiencies that were unveiled by the scholars reared its ugly head during the COVID-19 disaster response (Padayachee et al. 2020). The fact that the DMA was promulgated because of the severe increase in climate-induced disasters (Harrison 2009; Humby 2012; Padayachee et al. 2020; Pelling & Holloway 2006; Republic of South Africa 2005; Sithole 2014), then with the COVID-19 disaster, it shows that it failed to consider health as a hazard with the potential of becoming a pandemic that can cause a disaster (Padayachee et al. 2020). In response to this gap in the DMA, the CoGTA Minister hastily developed a supporting regulation (see Table 1: 18 March 2020).

**TABLE 1:** COVID-19 regulations and guidelines issued in South Africa from March to May 2020.

Year	Legislations	Brief purpose
15 March 2020	DMA: Classification of a national disaster: COVID-19 (coronavirus)	NDMC classified COVID-19 pandemic a national disaster in terms of section 15(2)(aA), 23(1)(b), 23(8), 24(4)–(8) of the DMA. NDMC designated coordination of the disaster and implementation of contingency arrangements to the national executive. Organs of the state required to prepare and submit reports to the intergovernmental forums.
15 March 2020	DMA: Declaration of a National State of Disaster: COVID-19 (coronavirus)	CoGTA Minister declared a national state of disaster, as designated under section 3, 27(1)(2) of the DMA, authorising her as a Cabinet member designated by the President to declare a national state of disaster and make, issue and authorise the issue of regulations and directions to assist, protect and provide relief to the public, protecting property, combating disruption and other effects of the disaster. Her declaration followed the WHOs and NDMCs classification.
18 March 2020	Regulations issued in terms of section 27(2) of the DMA	CoGTA Minister issued regulations to prevent an escalation and mitigation of the effects of the disaster. It contains definitions related to the disaster, for example, COVID-19, quarantine, WHO, gathering and isolation which were not in the DMA. It mandates the release of resources, prevention and prohibition of gatherings, refusal of medical examination, treatment, places of quarantine and isolation, closure of schools and partial care facilities, suspension of visits, etc.
25 March 2020	Directions, Response to COVID-19 in the CoGTA Sectors	-
26 March 2020	Amendment, issued in terms of section 27(2) of the DMA	-
30 March 2020	Directions issued in terms of section 27(2) of the DMA	-
16 April 2020	Amendment of regulations issued in terms of section 27(2) of the DMA	-
20 April 2020	Amendment of regulations issued in terms of section 27(2) of the DMA	-
29 April 2020	Amendment of regulations issued in terms of section 27(2) of the DMA	-
7 May 2020	Amendment of Directions issued in terms of section 27(2) of the DMA	-
14 May 2020	Once-off movement, Directions issued in terms of section 27(2)(f) of the DMA	-
28 May 2020	Determination of alert levels and hotspots, issued in terms of section 27(2) of the DMA	-

*Source*: Republic of South Africa, 2020, *Dismissal of Freedom Front Plus Disaster Management Act court challenge, Parliament*, viewed from https://www.parliament.gov.za/press-releases/dismissal-freedom-front-plus-disaster-management-act-court-challenge.

DMA, Disaster Management Act; NDMC, National Disaster Management Centre; CoGTA, Cooperative Governance and Traditional Affairs; WHO, World Health Organisation.

Observed is the DMAs ‘problematic placement’ of the disaster management function as uncovered by Van Niekerk (2014), the Minister of CoGTA’s decisions contradict the President’s. As precisely stated by Padayachee et al. (2020), there were worrying contradictions in communication between the CoGTA Minister and the President, as well as other Cabinet members (Singh 2020). For example, the contradictions were the decisions regarding the banning and unbanning of cigarette sales (De Vos 2020). If the disaster management function was in the Presidency Office, then there will be one voice and fewer contradictions because the Minister in the Presidency would be responsible (Van Niekerk & Du Plessis 2020).

Opposing political parties were vocal concerning the DMA, thus the Freedom Front Plus (FFP), Democratic Alliance (DA) and the Economic Freedom Fighters (EFF) (Padayachee et al. 2020). The FFP argued that the DMA is unconstitutional regarding the COVID-19 disaster declaration. Arguing that it does not have important safeguards against potential abuse of power, it does not provide for judicial oversight. There was confusion between the ‘state of emergency’ in the constitution and ‘national state of disaster’ in the DMA (Republic of South Africa 2020). The FFP attested that Section 23(8)1 of the DMA is unconstitutional, stating that the declaration of the COVID-19 disaster and the regulations were not scrutinised by a legislative oversight mechanism (Tandwa 2020). Therefore, the FFP proclaimed that the DMA should be amended to have similar provisions as the state of emergency that require a 60% vote in Parliament (Besent 2020). The DA concurs with FFP, that Section 27 of the DMA is unconstitutional and that the CoGTA Minister should lay before the Parliament, Section 27(2) before implementing the proposed regulations (Moosajee & Munga 2020). Concerning Section 1(b) and Section 7(2) of the DMA that make provision for the distribution of relief to those affected, the EFF realised that the DMA does not make provision for gender, race, youth and disability (Southern African Legal Information Institute [SAFLII] 2020).

The chronological trends of COVID-19 regulations and guidelines issued under the DMA by the CoGTA Minister are provided (Table 1).

After the first regulations were published by the CoGTA Minister, amendments to the regulation followed and led to a national lock-down. Since then, during the state of disaster, a deluge of other regulations was issued in terms of the DMA, section 27(3). This section stipulates that the state of disaster lapses 3 months after it has been declared and can be extended by one more month. However, the President has extended the state of disaster for the longest period in the history of disaster in South Africa (Staff Writer 2021).

The chronological issuance of regulations and regular communication during the national State of the disaster showed that the South African government was transparent, calm, reassuring, knowledgeable, capable and trustworthy (Singh 2020). This was an indication of good governance and constitutional democracy during the pandemic and must be adopted going forward. Mkhondo (2020) supported this postulation, arguing that direction-giving, meaning-making and empathy are vital traits a good leader has during crisis communication. Moreover, Singh (2020) assumed that the continuous revision of South Africa’s COVID-19 regulations shows the governments’ responsiveness to the citizens’ concerns. Therefore, the President, and the Health and CoGTA Ministers, demonstrated bold, assertive, courageous and decisive leadership through their strategies (Mkhondo 2020).

## Theoretical framework

The MPIP postulates that the government formulates policies to induce societal changes such as the establishment of institutions, new patterns or to change patterns in old institutions. The author Smith (1973) postulated that a government assumes that the formulation of policies automatically leads to implementation but that is not always the case. The reasons why the government does not always succeed are because of government bureaucracies, limited capacity, lack of qualified personnel, corruption, opposition to the policy itself, insufficient direction and control from politicians, tension, strain and conflict amongst the policy implementers’ and the society affected by the policy. The MPIP considers four components in implementing policies, which are: (1) the idealised policy, (2) the target group, (3) the implementing organisation and (4) environmental factors:

- The idealised policy is what the policymakers attempt to induce. It can be a formal law or programme that the government intends to implement. The law’s or programme’s success depends on matters like the degree and intensity of support the government is committed to implementing the policy and the needs and demands in society. Policies can be complicated or simple, they can be organisational or non-organisational and they can be distributive, redistributive, regulatory or emotive-symbolic. In this study, the idealised policy is the DMA, which reflected some of the idealised policies’ characteristics during the COVID-19 disaster and other disasters.
- The target groups are those expected to adopt the new patterns of interaction. These are the people within organisations or the groups that are most affected by the policy. The government expects the organisations and groups to change to meet the policies’ demands. There are certain factors to take into consideration, such as the degree of institutionalisation of the target group, the leadership and the prior policy experience of the target group. Because of COVID-19, the South African community becomes interest groups affected by the DMA and regulations imposed by the government.
- The implementing organisation is responsible for policy implementation. This organisation is usually a government bureaucratic unit. When implementing the policy, the organisation takes into consideration the administrative structure, the personnel’s qualifications, the leadership style and nature of the administrative organisation and the attention and care to meet the objectives of the programme. The implementing organisation of the DMA under investigation in this study is the metropolitan disaster management centre. It is the local event coordinator and supporter of the provincial and NDMCs. The DMA enactment led to the establishment of disaster management institutions at national, provincial and municipal spheres of government.
- Environmental factors are differing cultural, social, political and economic conditions that influence the implementation process vice versa. The implementation process is affected by tensions that occur both within and between the four components of the policy implementation process. Examples of discrepancies within the implementing organisation are insufficient personnel or lack of policy implementation skills. Tension can occur between the target group and the idealised policy, where the target group is not conforming to the new policy. We observe this between CoGTA and the public or various stakeholders. In conclusion, the implementation process generates feedback that might generate tension. The feedback may be supportive or disruptive to the new patterns and institutions brought about by the policy implementation.

It was of interest to this study to obtain the implementing organisation’s feedback regarding the DMA. Smith (1973:209) postulated that the functioning of established local governments can evoke tension from some ruling leaders at the national level who feel that the central government should control local units. He states that these new tensions might lead to the reshaping of governmental policy and the issuance of instructions to administrative units to exercise more control over local government. An observation and clear thought through Smith’s postulations relate entirely with the various scholars’ critic of the DMA. With the occurrence of COVID-19, Smith’s (1973) model predicted the public reaction to the legislation.

## Study area and methodology

This was a cross-sectional study of the metropolitan municipalities of South Africa targeting the disaster management officials in each municipality. The study purposively selected all the metropolitan municipalities in South Africa and then randomly selected disaster management officials from each metropolitan municipality. During the COVID-19 disaster, several of the metropolitan municipalities were declared virus hotspots. A qualitative approach using an interview guide gained insight into the participants’ perceptions of the DMA before and after the COVID-19 disaster declaration (Cooper & Finley 2014; Jaison 2018). A detailed systematic examination of relevant literature informed the interview guide.

The researchers called and emailed approximately 27 individuals from the eight metropolitan disaster management centres to invite them to participate in the study. On agreeing to participate in the study, the researchers sent the individual respondents a Blackboard Collaborate virtual link, where the researchers conducted the interview discussions. Blackboard Collaborate is a virtual classroom for online teaching and web conferencing (Blackboard 2020). The researchers had in-depth interviews with a total of 10 disaster managers from three metropolitan disaster management centres. From the 10 respondents, the study managed to obtain perspectives from seven males and three females. The rest of the disaster management personnel indicated that they could not participate in the study because of time constraints as they were responding to the COVID-19 disaster.

In support of the sample size, Dworkin (2012) postulated that a qualitative study that uses in-depth interviews, a sample of five to 50 or 25 to 30 participants can be sufficient to reach saturation and redundancy. Other scholars have also suggested varying sample sizes, from as little as two to over 400 (Fugard & Potts 2005). Scholars like Vasileiou et al. (2018) suggested that a qualitative study should not conduct more than 50 interviews if they want to manage the complexity of analysing. Ultimately, data saturation, data no longer revealing or yielding new themes, redundancy of data and the information power should inform the sample size for a qualitative study.

## Empirical findings and discussion

This section presents the analysed and interpreted data in various categories that emerged from the virtual interviews. To add to the validity and richness of the results, some quantitative data are interlinked with the qualitative data. From the eight municipalities, four were from Buffalo City, four from Ekurhuleni, two from the City of eThekwini and the rest had no respondents.

### Implementing the legislation

All the respondents agreed that they are qualified enough to implement the DMA. The respondents’ tertiary education could be the reason for the positive response. When implementing policy, The Model of Policy Implementation Process suggests that the responsible organisation must have qualifications and the confidence to meet the objectives of the programme. Work experience and practical exposure add to the comprehension and ability to implement policy. In a study conducted in 2017 by Wentink and Van Niekerk, (2017), they found that only 41.2% of metropolitan municipalities personnel had tertiary education. This study found that all the respondents do have a tertiary education.

### Respondents perceptions of the legislation before COVID-19

Most of the respondents, six, were aware of the various scholars’ critique of the DMA, and the rest, four, were unaware. All the respondents who were knowledgeable of the critics agreed with the academics (see Table 2). They explained that the wording in the DMA leads to confusion, misinterpretation and leads to a lack of accountability that leads to non-implementation. The placement of the disaster management function exposes them to excessively complicated administrative procedures and insubordination making it difficult to implement it. Without stating specifics, a respondent disagreed with some of the funding models recommended, stating that they do not apply to the local context.

**TABLE 2:** Responses to the question, ‘Are you aware of scholars’ critique of the Disaster Management Act and the function?’

Critiques	Perceptions
Section 43, the establishment of municipal disaster management centres. Sub-section 43(2)(b) may operate such centre in partnership with those local municipalities	If it is ‘may’, it gives the impression that the district can choose to operate with other stakeholders or not. Our function is in the office of the general managers and there is a bureaucratic tape of reporting
Prof. D. Van Niekerk’s critic of the funding models	I agree with the content of his findings on funding models, but I do not entirely agree with the recommendations because some aspects do not apply to the South African context
Placement of the disaster management function in the highest political office. The DMA does not compel the placement of municipalities in the highest political office	There is a red tape, for example, in my municipality before a decision can be made, four signatures are needed. During a disaster, there is no time to find signatures because each minute you waste, you are losing life
The DMA is good on paper and not implementable	The scholars are right; I have noticed that the responsibilities are clear in the DMA. For example, the disaster management function is to coordinate. However, the challenge is the placement of the disaster management centre, which is at the same level as other departments. Therefore, other departments do not take disaster management seriously when it comes to giving other departments instructions.
There is inadequate funding for disaster management activities	The funding is not sufficient to implement disaster management activities, especially if a disaster is not declared.

DMA, Disaster Management Act.

A significant number of disaster managers are knowledgeable of the scholar’s perceptions of the legislation. Academics’ work of reviewing and analysing policies is needed in a democracy. However, their work sometimes leads to turmoil, but the quality of governance is improved (Kumar 2019).

### Difficulties experienced when implementing the legislation

All the respondents indicated difficulties experienced when consulting the DMA for their disaster management operations. The identified problematic sections in the DMA that they identified as problematic sections are listed (Table 3).

**TABLE 3:** Problematic sections in the legislation.

Sections	Brief description
Section 2(1)(b)(ii)	Application of the Act does not apply to an occurrence that can be dealt with effectively in terms of other national legislation. The Minister gazettes the occurrence.
Institutional arrangements sections	Disaster management role-players in the municipality, for example, traditional leaders, businesses, etc.
Section 23 and Section 55	(23) Classification and recording of disasters, (55) Declaration of a local state of disaster
Section 51	Municipal disaster management advisory forum
Section 17(2)	Disaster Management Information System
Section 51,52,53	(51) Municipal disaster management advisory forum, (52) Preparation of disaster management plans by municipal entities, (53) Disaster management plans for municipal areas
Section 44(1)(e)	Powers and duties of a municipal disaster management centre
Section 56	Funding of post-disaster recovery and rehabilitation guiding principles

Section 2(1)(b) was identified as a difficult section that leads to friction between disaster managers and sector department managers at the same levels. The respondent explained that some sector departments refuse to use their legislation to obtain funding from disaster management once the event is declared a disaster. They further argued that to make the situation worse, the CoGTA Minister never gazettes emergencies. Concerning institutional arrangements, a respondent revealed that the stakeholders they are supposed to work with expect the disaster management function to do everything. Yet, the function of the disaster management portfolio is coordinating activities (Republic of South Africa 2002). Therefore, the sections on committees and institutional arrangement in the DMA should be clear and specific on the responsibilities of the stakeholders. Concerning Section 23, a respondent asserted that the NDMC is not transparent with the information and procedures on disaster classification, which in turn delays their operations. This could be attributed to the lack of clear guidelines on the process to be followed when classifying and recording disasters. The respondent further stated that Section 23 and Section 55 confuse them because Section 23 indicates that ‘…the National Centre must immediately classify the disaster as a local, provincial or national disaster…’, whilst Section 55 says:

[*I*]n the event of a local disaster, the council of a municipality having primary responsibility for the coordination and management of the disaster may, by notice in the provincial gazette, declare a local state of disaster (Republic of South Africa 2002:54)

The respondent’s confusion was who has the final power to declare a local state of disaster, National or Council? Another respondent referred to difficulties with Section 51 on municipal disaster management advisory forums that the involvement of politicians like the Mayor in such meetings poses challenges. The respondent further argued that politicians attend these meetings with personal agendas of gaining favour during disaster relief interventions. In the South African context, Mayors are political appointees who serve on a political ticket and therefore, carry the mandate of fulfilling a political agenda. This manifests in disaster relief activities, whereby the interventions do not comply with the basic human rights of saving lives but instead the interventions are biased towards the ruling party.

Another difficulty mentioned by a respondent was Section 17(2) concerning the development of an electronic database with extensive information on disaster management issues by the NDMC. The respondent pointed out the fact that the NDMC takes a long time to provide information when requested. Mention was made by a respondent that whilst the DMA (Section 51, 52, 53) mandates that all disaster management role players in the municipality must attend advisory forum meetings and develop disaster management plans; unfortunately, the role-players do not attend or prepare the plans. To try and encourage or obligate the sector departments to attend advisory forum meetings and to have operational disaster management plans, these activities were included in the HOD’s scorecard. This has improved the attendance and the provision of disaster management plans in the municipality. The HOD makes it mandatory for everyone to take disaster management activities seriously. To deal with the lack of attendance and plans, the respondent indicated that the Head of Department’s scorecard is affected. There is a respondent who said that concerning Section 44(1)(e) on the funding of disaster management in the municipal area, it does not ensure that there is funding, the sections state that the municipality makes recommendations, which is not enough. The respondent further argued that Section 56 only focusses on post-disaster funding and not pre-disaster funding. This makes the DRR activities difficult to undertake in the municipalities, thereby making communities more vulnerable to impending hazards. Several scholars have identified the DMA as the legislation that translated the management of disasters from response and civil protection to risk reduction (Chagutah 2014; Humby 2012; Jordaan 2018; Van Niekerk 2014). On the contrary, Section 56 somehow opposes the shift from reactive to proactive with its clear mention of ‘funding of post-disaster recovery and rehabilitation guiding principles’.

### Perceptions of Section 3 and 27(1) before COVID-19

Central to the investigation of the study was the disaster managers’ perceptions of the infamous Section 3 and 27(1) that were used to declare COVID-19 a disaster before the disaster declaration. Section 3 empowers a Cabinet member designated by the President to administer the DMA. Section 27(1) empowers the Minister to declare a national disaster. Some respondents said that Section 3 should be assigned to the office of the President and not a Minister. Other respondents mentioned that Section 3 should be designated to the NDMC rather, ‘I do not think politicians should administer policies, they politicise everything’ said the respondent. Section 3 is problematic because of the different views on who should administer the legislation. Regarding Section 27(1), the respondents were not comfortable with the wording that the Minister ‘may’ declare a national disaster.

### Perceptions of the legislation and the function after COVID-19

Most of the respondents, seven, were knowledgeable of the public’s critiques of the DMA and the disaster management functions, and the rest, three, were unaware. The respondents were knowledgeable of the public’s critiques like the unconstitutionality and unaccountability of the NCCC, poor implementation of the DMA with both the Minister and the President declaring a disaster, lockdown unconstitutional, the CoGTA Minister was given too much power and challenged in court and the business society complaining about the loss of income because of the regulations imposed. Most of the respondents disagreed with the critiques, ‘The critique of the establishment of the NCCC and its unconstitutionality was misinterpreted and based on misinformation. It was a very inclusive structure informed by the specialists’. The respondents argued that the inclusion of various representatives on the NCCC was good as it allowed for scientific and social deliberations and also transparency and accountability of the decisions made. Another respondent said, ‘I disagree with the critic on lockdown being unconstitutional because the regulations were implemented to save people lives’, correlating to the disaster managers’ coordination role (Republic of South Africa 2002).

The implementation of Section 3 and Section 27(1) during the COVID-19 disaster revealed mixed perceptions. The respondents argued that Section 3 designates the Minister of CoGTA to administer the DMA, but empowering other Ministers caused confusion. There was limited oversight and the disaster was politicised, argued one respondent. Some of the disaster managers believed that the NDMC should be responsible for the administration of the DMA as they were actively advising the Minister. Concerning Section 27 (5), on the declaration of a national disaster, which lapses after 3 months allowing the Minster to terminate or extend the declaration with 1 month, a respondent contended that this section does not apply to a long-term indefinite biological disaster like COVID-19. It is according to the DMA that the disaster was extended seven times, from the declaration day to 15 July to 15 August, to 15 September, to 15 October, to 15 November, to 15 December 2020 and to 15 January 2021.

### Respondents roles and responsibilities in response to COVID-19 and challenges

To better understand the challenges, disaster managers encountered whilst implementing the DMA during the COVID-19 disaster, the study examined their roles and responsibilities. Their roles included the coordination of, for example, South African Police Services (SAPS) and Health. They ensured compliance with COVID-19 regulations at places like funerals. COVID-19 public awareness, education and the monitoring of infection progression were some of the critical roles. They developed plans informed by the regulations and distributed relief, for example, medical supplies. For reporting and decision-making, the disaster managers actively participated in committees such as the municipal COVID-19 command council and provincial joint operation committee. The coordination role of the disaster managers as prescribed in the DMA is observed. Regardless of the legislation’s prescription, the disaster managers indicated challenges experienced when operating. Challenges included the lack of compliance, ‘we would advise the SAPS of non-compliance at funerals but they do not issue the fines’, and ‘sector departments do not give us feedback reports on COVID-19 activities like installation of sanitisers’. This points to the earlier finding on the placement of the function, which, in some instances, calls into play insubordination. Van Niekerk’s (2014) study on the critical analysis of the DMA found that some stakeholders lacked understanding of the disaster management function. He also found a lack of cooperation and involvement by other government departments. Considering the way other stakeholders view the disaster management function, a disaster manager revealed that they serve as a participant and not a coordinator in the COVID-19 Municipal Council. This strongly revealed the other stakeholders’ lack of oversight of the disaster management function.

### Level of trust in the legislation and its use as a guide for responding to COVID-19

Despite the criticism of the DMA by the academics and the public, which burgeoned because of the COVID-19 disaster, six of the disaster managers indicated that their trust in the DMA was ‘very high’. The trust of other disaster managers, three in number, was ‘high’, and just one indicated that they are now ‘doubtful’. The Policy Implementation Process Model postulates that those implementing policy experience tensions, strains and conflict. Whilst the COVID-19 regulation implementation brought about these experiences, the disaster managers still hold the DMA with high regard.

Most, six, of the disaster managers perceived the DMA as a ‘very useful guide’ for managing COVID-19 and the rest, four, saw it as a ‘useful guide’. ‘The DMA was a useful guide for declaring the COVID-19 virus a disaster because immediately after the declaration, resources were released and many other activities relating to the disaster implemented’, said a respondent. The positive perception relates to the belief that the DMA is one of the best disaster management legislations in the world (Davis 2015; Humby 2012).

### Perceptions of the Minister of Cooperative Governance and Traditional Affairs

Mr John Steenhuisen, the interim leader of the opposition party DA, criticised the CoGTA Minister for failing to implement sound logic when announcing some regulations (Polity News 2020). ‘She was criticised a lot from the political sphere’, said a respondent. In response to the question of how the respondents perceived how the CoGTA Minister represented the function in response to COVID-19, most, nine, of the respondents felt that she represented them well.

‘I believe the Minister has represented the disaster management fraternity well as she is strict and firm with her decisions.’ (Participant 1, male, > 55 year olds)

‘The priority was to save lives, regardless of the economic suffering, which she later focused on.’ (Participant 2, female, 31–42 year olds)

‘Even though she was criticised a lot by the political sphere, we need MEC’s and Mayors like her.’ (Participant 4, male, 31–42 year olds)

‘She is doing her level best through the NDMC guidance and provides other Ministers with a chance to deal with their portfolios.’ (Participant 5, female, 31–42 year olds)

On the contrary, one respondent believed that the NDMC was supposed to lead instead of the CoGTA Minister because she is a political figure.

### Policy implementation process and the Disaster Management Act

With the request to mention more than one option, a significant number of the respondents, eight, indicated that the DMA is an excellent piece of legislation. From these eight respondents’, only two respondents indicated that it also causes conflict. Just as supported by the literature, no respondent indicated that the legislation is poorly constructed, but they indicated that it causes conflict, at least three, and the other two argued that it causes tension. ‘The DMA causes conflict because I do not have the authority to instruct other people when coordinating an incident’, argued one respondent. A respondent who selected the option, ‘it causes tension’, said, ‘It is a good legislation, but I feel like there are challenges in implementation’. Tension is a strained relationship between the disaster managers and society, for example, politicians or amongst the disaster managers themselves, the national level and the local level.

## Conclusion

This article discussed the disaster managers’ perceptions of the DMA before the COVID-19 disaster declaration in the literature review and after, based on the empirical research. The aim was to provide the disaster managers with an opportunity to share their opinion of the academics and public critic of the legislation that mandates their operations. Recapitulating the scholars’ critical analysis of the DMA was important for this study. The gaps the scholars identified had a significant impact on the response to the unanticipated pandemic. Observed is the need for disaster managers to consider academic research for improving their practices and advocate for the review of the DMA. Even though the DMA was heavily criticised, the disaster managers’ faith in the idealised legislation was not tested but rather presented them with a different perspective. Despite their impressive trust and respect for their legislation, the governance of the institution fails to support the effective implementation of the DMA. Many postulations by Smith (1973) about the implementation of policies are proved correct in this study. The MPIP was significant because of its grounding in policies. Whilst policymakers can consider applying the MPIP, there is no guarantee that its application will mitigate disruptive tensions that fail the outcomes of the policy to meet needs. Ultimately, by emphasising and echoing various scholars’ significant findings on the difficulties the disaster managers encounter in the governance of the disaster fraternity, the study hopes the South African government to improve this institution. In the face of the study findings, this study provides the practical recommendations:

- Disaster management centres must employ researchers who can update the practitioners on the scholarly and public perceptions of the disaster management policies through advisory forum meetings and other platforms. The disaster managers’ conference attendance must be mandatory.
- COVID-19 exposure of some gaps in the DMA, which frustrated the disaster managers, should be a rude awakening for the NDMC to review the legislation for it to be inclusive of biological hazards and provide the disaster management fraternity with more authority.
